# Large-scale transport of PM_2.5_ in the lower troposphere during winter cold surges in China

**DOI:** 10.1038/s41598-017-13217-2

**Published:** 2017-10-16

**Authors:** Jianjun Wang, Meigen Zhang, Xiaolin Bai, Hongjian Tan, Sabrina Li, Jiping Liu, Rui Zhang, Mark A. Wolters, Xiuyuan Qin, Miming Zhang, Hongmei Lin, Yuenan Li, Jonathan Li, Liqi Chen

**Affiliations:** 1 Key Laboratory of Global Change and Marine-Atmospheric Chemistry, Third Institute of Oceanography, State Oceanic Administration, Xiamen, China; 2 State Key Laboratory of Atmospheric Boundary Layer Physics and Atmospheric Chemistry, Institute of Atmospheric Physics, Chinese Academy of Sciences, Beijing, China; 3 State Key Laboratory of Marine Environmental Science, and College of Ocean & Earth Sciences, Xiamen University, Xiamen, China; 4 Department of Geography and Environmental Management, University of Waterloo, Waterloo, Canada; 5 Department of Atmospheric and Environmental Sciences, University at Albany, State University of New York, Albany, USA; 6 Shanghai Center for Mathematical Sciences, Fudan University, Shanghai, China

## Abstract

A comprehensive investigation using the air quality network and meteorological data of China in 2015 showed that PM_2.5_ driven by cold surges from the ground level could travel up to 2000 km from northern to southern China within two days. Air pollution is more severe and prominent during the winter in north China due to seasonal variations in energy usage, trade wind movements, and industrial emissions. In February 2015, two cold surges traveling from north China caused a temporary increase in the concentration of PM_2.5_ in Shanghai. Subsequently, the concentration of PM_2.5_ in Xiamen increased to a high of 80 µg/m^3^_,_ which is double the average PM_2.5_ concentration in Xiamen during the winter. This finding is a new long-range transport mechanism comparing to the well-established mechanism, with long-range transport more likely to occur in the upper troposphere than at lower levels. These observations were validated by results from the back trajectory analysis and the RAMS- CMAQ model. While wind speed was found to be a major facilitator in transporting PM_2.5_ from Beijing to Xiamen, more investigation is required to understand the complex relationship between wind speed and PM_2.5_ and how it moderates air quality in Beijing, Shanghai, and Xiamen.

## Introduction

China’s rapid economic development and urbanization in recent decades has resulted in major air pollution that has serious complications for the environment and human health^1,2^. Increasing haze and smog episodes caused by high concentrations of fine particulate matter (PM_2.5_, with aerodynamic diameters not larger than 2.5 μm) has significantly reduced visibility across many cities in China. In the megacity clusters of the Yangtze River Delta (YRD), the Pearl River Delta (PRD), and the Beijing-Tianjin-Hebei (BTH) regions, haze is a significant threat to human health as it is linked with increasing levels of morbidity and mortality^3–6^. The design of appropriate pollution reduction strategies requires knowledge of major pollution sources, emission levels, pathways and timescales of intercontinental air pollution transport dispersal and deposition processes, and the atmospheric processes of PM_2.5_. Understanding the transport mechanisms of air pollutants is essential for reducing regional air pollution.

Air pollution is more severe during the winter and more prominent in northern China, which is consistent with the monitoring data of the national air quality monitoring network^7^ and also the PM_2.5_ concentrations retrieved from a combined geophysical-statistical method with information from satellites, models, and monitors^8^. This is due to variations in geographical location, seasonal energy usage, influence of trade winds, and spatial distribution of industries. Xiamen is an island located in southeast China that has been recognized for its high air quality. In recent years, the island has experienced an increasing number of haze episodes^3,5^. The average PM_2.5_ in Xiamen is 39 µg/m^3^ in winter and 29 µg/m^3^ in summer. The arrival of cold surges from northern China could cause the concentration levels of PM_2.5_ in Xiamen to reach 80 µg/m^3^. We investigated data on wind field and air quality and observed that the deterioration of air quality in Xiamen was most likely due to long-distance transport of pollutants from northern China.

Pollutants emitted to the atmosphere from natural or anthropogenic sources may be transported for distances from several to hundreds of meters (micro-scale), through thousands of meters to hundreds of kilometers (meso-scale), to hundreds to thousands of kilometers (macro-scale)^9–12^. The transport distance of the air pollutants mainly depends on meteorological conditions, such as wind direction and speed, and varies with the lifespan, deposition velocities, and altitude of the pollutants. Pollutants with longer lifespan and lower deposition velocities can reach higher altitudes within the air column and can be transported farther in distance. Most studies on the transport of air pollutants are based on computer-based models^9,13–15^, satellite monitoring^16–22^, or monitoring data from individual or networks of ground stations^6,15,23–27^. Literature has generally acknowledged that air pollutants will be carried farther in the upper troposphere than in the lower troposphere for long-range transport at the macro-scale^15,28^.

**Figure 1:**
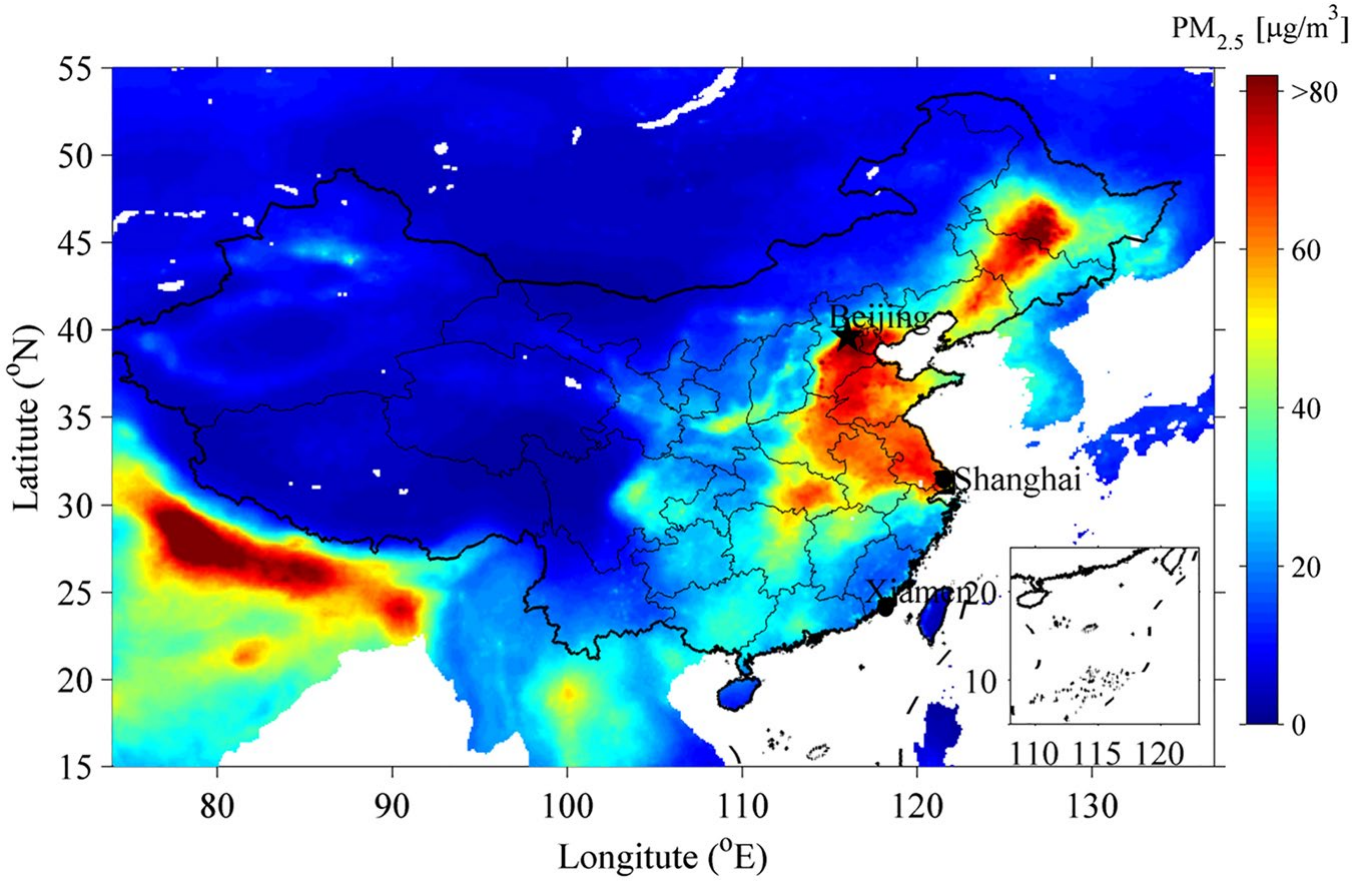
Map of China, and locations of Beijing, Shanghai and Xiamen. The ground-level PM_2.5_ is estimated by combining Aerosol Optical Depth (AOD) retrievals from the combined geophysical-statistical method with information from satellites, models, and monitors^8^. Figure is plotted using MATLAB R2013a (http://www.mathworks.com/).

PM_2.5_ concentrations higher than 80 μg/m^3^ were observed in Xiamen from 1 February to 10 February in 2015. Two fast-moving cold surges with maximum ground wind speeds over 20 m/s developed during the same time interval. The high speed of cold surges enabled the transport of pollutants from north China to south China before they can be deposited. In this study, we closely monitored the evolution and transport of air pollutants from north to south China during the time interval from1 February to 10 February 2015. The Hybrid Single-Particle Lagrangian Integrated Trajectory (HYSPLIT) model for back trajectory analysis^29,30^, the three-dimensional air quality modeling system, and the Regional Atmospheric Modeling System and the Community Multiscale Air Quality Model (RAMS-CMAQ)^22^ were used to track the southward transport progress of PM_2.5_. In addition, we used observations of hourly PM_2.5_ concentration and the wind field in Beijing, Shanghai and Xiamen to investigate the effect of wind speed on air quality. The distance between Beijing and Shanghai is about 1063 km and that between Shanghai and Xiamen is about 800 km (Fig. 1).

## Results

### Meteorological conditions during the study interval

The winter weather in mainland China is cold and dry, dominated by East Asian winter monsoon. The most prominent surface feature of the winter monsoon is cold surges induced by strong northwesterly winds along the eastern flank of the Siberian high, leading in turn to rapid temperature falls in southern China^31,32^. The major track of anticyclones in this season extends from the source region southeastwards, passing the lower part of the Yellow River, then eastwards through the Yellow Sea, Korean Peninsula, and then to Japan. As the winter progresses, this major track shifts southeastwards to the coast of the East China Sea^33^.

In February 2015, two cold surges developed during a ten-day study interval. The first cold surge appeared on 3–6 February and the second cold surge appeared on 6–9 February; both surges spread from Beijing to Xiamen. The first cold wave weakened during the southward movement; peak wind speed in Beijing was 25 m/s at 03:00 on 3 February and 16 m/s at 11:00 on 5 February in Xiamen. In contrast, the second cold wave strengthened during its movement south; the peak wind speed in Beijing was 22 m/s at 19:00 on 7 February, 3:00 on 8 February in Shanghai and 21:00 on 8 February in Xiamen (Table 1). The lack of precipitation during the two cold surges made the investigation of long-range transport of PM_2.5_ possible.

**Table 1:** The wind speed and the PM_2.5_ concentrations in Beijing, Shanghai and Xiamen during the two cold surges

	Beijing	Shanghai	Xiamen
**The first cold surge**			
Strong wind > 8 m/s period	16:00 on 3^th^ − 04:00 on 5^th^	08:00 on 4^th^ − 20:00 on 5^th^	19:00 on 5^th^ − 23:00 on 6^th^
Max of wind speed	25 m/s	20 m/s	16 m/s
Variation of PM_2.5_	Decreased from 212 μg/m^3^ to concentrations lower than 20 μg/m^3^	Increased from 50 μg/m^3^ to 278 μg/m^3^, then decreased to 60 μg/m^3^ afterwards	Increased from 20 μg/m^3^ to around 80 μg/m^3^
**The second cold surge**			
Strong wind > 8 m/s period	16:00 on 7^th^ − 14:00 on 8^th^	02:00 on 8^th^ − 15:00 on 9^th^	09:00 on 8^th^ − 20:00 on 9^th^
Max of wind speed	22 m/s	22 m/s	22 m/s
Variation of PM_2.5_	Decreased from 47 μg/m^3^ to concentrations lower than 10 μg/m^3^	Increased 88 μg/m^3^ to 164 μg/m^3^, then decreased to 9 μg/m^3^	75–81 µg/m^3^ during the interval of high wind speed

### Pollutant transport during the cold surges

**Figure 2:**
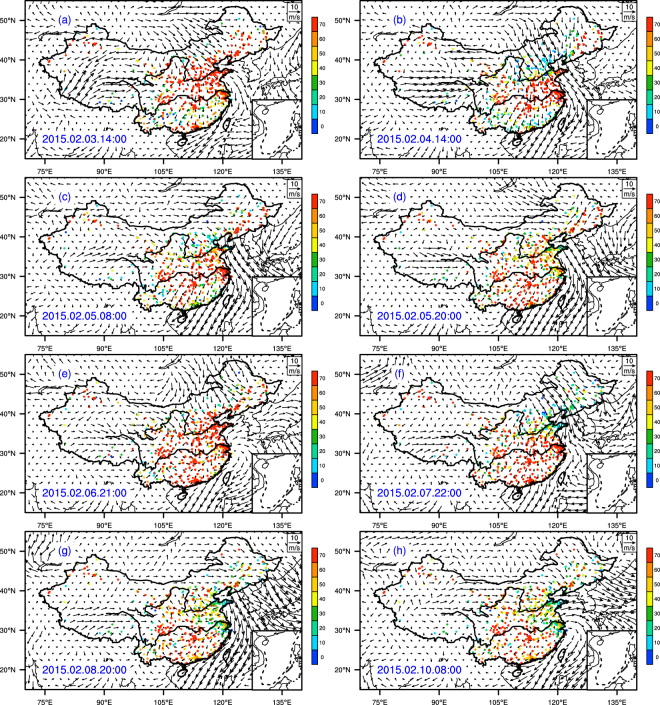
Temporal and spatial distribution of the wind field and concentrations of monitoring PM_2.5_ from 1 to10 February, 2015. The figure is created by open access software of the NCAR Command Language (Version 6.4.0, [Software]. (2017). Boulder, Colorado: UCAR/NCAR/CISL/TDD. http://dx.doi.org/10.5065/D6WD3XH5).

**Figure 3:**
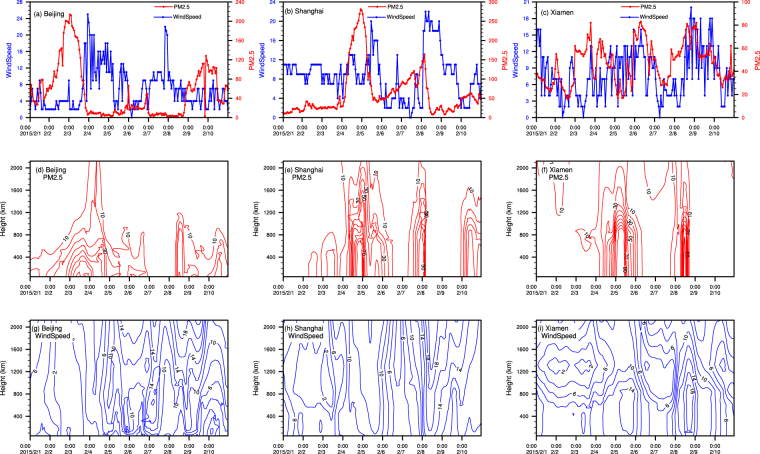
Time series of *in situ* wind speed and concentrations of PM_2.5_, and simulated vertical structure of PM_2.5_-height and wind speed-height extracted from RAMS-CMAQ for Beijing, Shanghai and Xiamen during 1–10 February 2015. The figure is created by open access software of the NCAR Command Language (Version 6.4.0, [Software]. (2017). Boulder, Colorado: UCAR/NCAR/CISL/TDD. http://dx.doi.org/10.5065/D6WD3XH5).

The spatial distribution of PM_2.5_ concentration and the wind field (ERA-Interim) over mainland China during 1–10 February 2015 is illustrated in Fig. 2. During the beginning of the first cold surge, hourly PM_2.5_ concentrations exceeded 75 over μg/m^3^ in northern China, while concentrations in mega cities of northern China exceeded 200 μg/m^3^. The first cold surge then moved to central China by 8:00 on 4 February and to southern China by 20:00 on 5 February. The second cold surge developed on 6 February and was dispersed in northern China at 21:00 on 6 February. The second cold surge passed central China by 06:00 on 8 February and reached south China by 20:00 on 8 February (Table 1 and Fig. 2). An animation of the spatial distribution of PM_2.5_ and the wind field in China during 1–10 February can be accessed via Supplemental Materials 1. The detailed concentrations of PM_2.5_ and wind speed in Beijing, Shanghai, and Xiamen are illustrated in Figs 2 and 3 and also listed in Table 1.

### The back trajectory for Xiamen during the heavy pollution episodes

**Figure 4:**
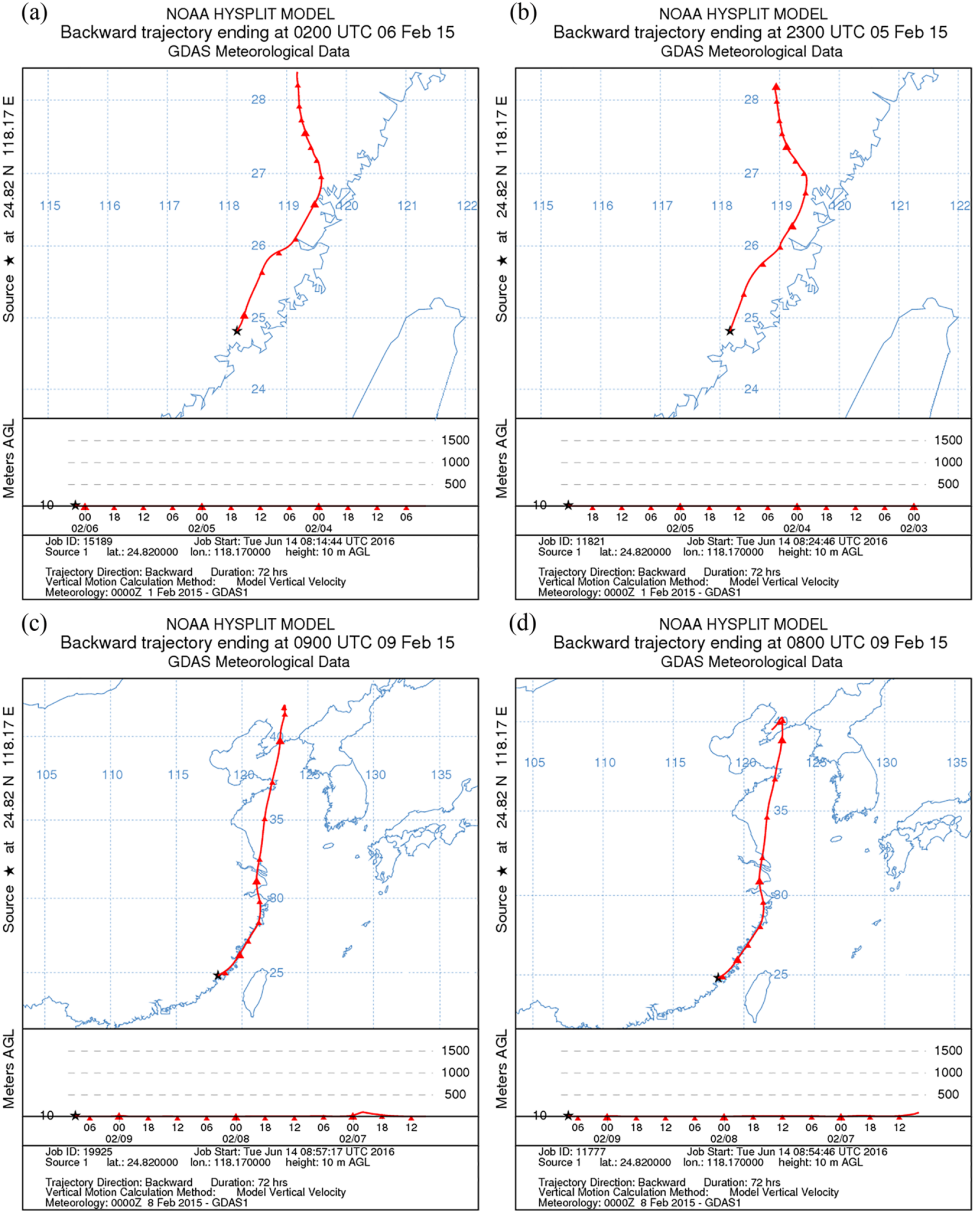
Back trajectories for Xiamen during the interval of severe air pollution. Figures are plotted using HYSPLIT Trajectory Model provided by Air Resources Laboratory of NOAA^29,30^.

In order to investigate the origin of air masses arriving in Xiamen during the study interval, the isentropic 3-day back trajectories ending at 10 m in Xiamen were computed using the HYSPLIT model. During the episodes of severe air pollution in Xiamen on 6, 8 and 9 February, it can be observed that ground level air masses came from north China (Fig. 4). Two main transport pathways for pollutants were observed during the cold surges; the pollutants travelled directly southwards to south China and southwestwards to the Yellow Sea, then southwestwards along the coast of East China Sea to South China. Pollutants were sourced from heavy polluted provinces such as Shandong, Henan, and Liaoning. The back trajectories agreed reasonably well with the *in-situ* air quality monitoring network of China and the RAMS-CMAQ model simulations. The findings of this study are partly consistent with another research study that used the trajectory model, which found that the concentration of dust parcels from south China Sea are within a thin layer at less than 200 hPa, before descending to Taiwan^5^.

### Reproduction of the transport of air pollutants during the two cold surges using the RAMS-CMAQ models

**Figure 5:**
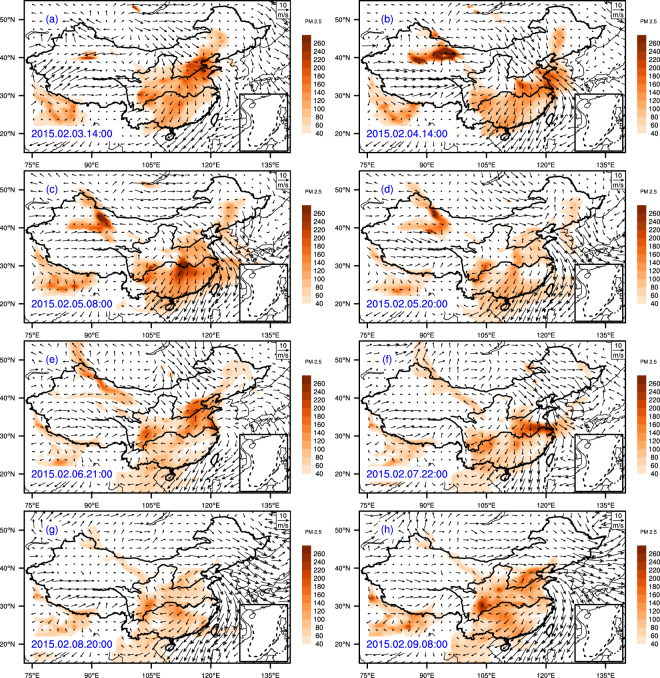
Modeled transport of PM_2.5_ at 50 m altitude during 1–10 February 2015 by RAMS-CMAQ model. The figure is created by open access software of the NCAR Command Language (Version 6.4.0, [Software]. (2017). Boulder, Colorado: UCAR/NCAR/CISL/TDD. http://dx.doi.org/10.5065/D6WD3XH5).

Our models were able to reproduce the movement of pollutant transport during the two cold surges in February 2015 (Fig. 5). The model illustrates the movement of the first cold surge from the northern region to the central region (4 February), and from the central region to the southern region (6 February). The second cold surge started in the north during the night of 7 February, moved to central China on 8 February, and then to south China on 8 and 9 February. Time series of height-PM_2.5_ concentrations and height-wind speed were also extracted from the model (Fig. 3). Since *in-situ* monitoring stations were located only at ground level, model estimates of variations in PM_2.5_ concentration, wind field, and height were studied to understand the vertical structure of the transport progress.

## Discussions

### The complex effect of wind on pollutant transport in different cities

Existing literature has recognized that wind speed is negatively associated with PM_2.5_^34,35^. Therefore, the increase of the wind speed would result in decrease in the concentration of PM_2.5_. However, the correlations between wind speed and air pollutant concentrations in Beijing, Shanghai, and Xiamen during the two cold surges reveal a more complex relationship. Based on the results of RAMS-CMAQ model, the concentration of PM_2.5_ in Beijing shared a negative correlation with wind speed (Fig. 4), such that a high concentration was evident during an interval of low wind speed, and vice versa. In contrast, a high concentration of PM_2.5_ was observed in the model during an interval of high wind speed in Xiamen, showing that the correlation between PM_2.5_ and wind speed was positive. In Shanghai, our model showed that a high concentration of PM_2.5_ was initially evident, but decreased over time during the interval of high wind speed.

Ambient PM_2.5_ concentration can be influenced by the interaction of wind with local and regional industrial emissions. Harrison *et al*.^36^ divided the particle fraction into two components: a non-wind-suspended component derived from industrial and construction activities, traffic-induced re-suspension, and biological particles, and a wind-suspended component from natural sources such as sea spray, surface soils, or dusts in paved areas, and find an increase in mean wind speed can facilitate both mechanical formation of the particles and their entrainment.

This is consistent with the findings of many previous studies in Beijing and other cities^37^. High wind speed can reduce the concentration of PM_2.5_. Dilution and dispersion due to wind can potentially result in a 30% reduction in the concentration of PM_2.5_, as seen in London during both weak and strong winds^38^, and a similar dependence on wind speed for PM_2.5_ was also observed in Birmingham^39^. In addition, a statistically significant (p ≤ 0.01) negative correlation between PM_2.5_ and wind speed during the winter and summer seasons was observed in Islamabad, Lahore, Peshawar and Quetta in Pakistan^35^. When wind speed is low, local emissions can substantially influence the concentrations of PM_2.5_. However in our study, it was found that wind speeds exceeding 10 m/s resulted in the removal of almost all pollutants from the northern cities during the two cold surges.

Compared to Beijing, Xiamen has less industrial development, lower population density, and thus lower energy consumption. Xiamen is an air pollutant “sink” or receptor city, which means that strong winds can transport air pollutants from other cities to Xiamen. For this reason, there was a surge in the concentration of PM_2.5_ in Xiamen during the interval of strong winds. This result shows that cities in southern China are influenced by the concentration of air pollution and wind conditions in northern China. To some extent, wind speed is a factor that can be used to forecast the deterioration of air quality in southern Chinese cities during cold surges.

However in the case of Shanghai, which is located between the source and receptor city, the interaction between wind speed and pollution coming from northern cities is more complex and less straightforward. During the two cold surges, the concentration of pollutants in Shanghai increased with increased wind speed during the first two hours, then decreased with increased wind speed for the remainder of the time. The decrease in the concentration of PM_2.5_ could be due to the wind field, concentration of pollutants from source cities, and the initial concentration of pollutants in the city and adjacent to the city. Since the spatial distribution of PM_2.5_ concentrations in China consisted of higher concentrations in the north and lower concentrations in south, the concentration of PM_2.5_ in central China is approximately the average between the concentrations in the north and south. The impact of winds on PM_2.5_ concentrations in Shanghai is also intermediate between its degree of impact on the northern and southern cities. This is the first time such phenomenon has been observed.

### Discrepancies between RAMS-CMAQ and the *in situ* monitoring network

Although RAMS-CMAQ simulated movement patterns of two pollutant transport episodes, there were minor discrepancies between the results obtained from the model and *in-situ* monitoring measurements. During the ten-day monitoring interval, the maximum PM_2.5_ concentrations exceeded 200 μg/m^3^ for Beijing and Shanghai, as recorded by *in-situ* measurements, which was underestimated by the simulation with 70 μg/m^3^ for Beijing and 110 μg/m^3^ for Shanghai, respectively. The maximum PM_2.5_ in Xiamen was 77 μg/m^3^ according to *in-situ* measurement, which was overestimated by the model with 110 μg/m^3^. There were also differences in wind speed; for example, the maximum *in-situ* wind speed was 18 m/s at 16:00 on 3 February in Beijing, while the modeled wind speed was less than 4 m/s at that time, and reached 10 m/s at noon on the following day. The maximum *in-situ* wind speed was 20 m/s at 09:00 on 5 February in Shanghai, while the modeled wind speed was around 12 m/s at that time. Similar underestimation of the wind speed also occurred during the first cold surge in Xiamen, with the *in-situ* wind speed exceeding 15 m/s on 6 February, while the modeled value was 10 m/s. RAMS-CMAQ has been widely used to model and predict air quality in China. The discrepancies between the meteorological and emission datasets for RAMS-CMAQ may have contributed to the discrepancies between the RAMS-CMAQ model and *in-situ* measurements.

There were also the temporal shifts between observations and simulations. For Beijing, the PM_2.5_ concentration first peaked around 0:00 on 3 February, almost 12 hours before it peaked in the RAMS-CAMQ simulation; while PM_2.5_ peaked around 5:00 on 6 February, 20 hours later than it peaked in the simulation for Xiamen. The main reason for the phenomena might be due to the underestimation of the accumulate rate of PM_2.5_ in the model under the low wind speed, and the complexity of land surface, and the frictional force consequently, which leading to the overestimation of the PM_2.5_ transport speed with the cold surges.

### Relationship of PM_2.5_ concentration and altitude in long range transport

The modeled PM_2.5_ concentrations decreased with increasing altitude, which is consistent with many previous observations. The results of a previous study conducted in Beijing indicated that PM_2.5_ concentrations at 320 m altitude were about 29% and 78% of those at 8 m altitude during days with good and poor air quality, respectively^16^. Horvath *et al*. found that the mass concentration of diesel combustion-derived particles at 27 m altitude was 83% of that at street level ^19^. Chan and Kwok’s research suggested that the relationship between the decrease in the mass concentration of particles was exponential in a street canyon and linear at open sites^40^.

In general, literature has indicated that in the case of long distance transport, such as at an intercontinental scale of ten thousand of kilometers, pollutants were more likely to be transported at high altitude than at low altitude^15,41^. The results of the FLEXPART model demonstrated that Asian tracer was pumped selectively into the upper troposphere, as opposed to a diffusion-like dispersion process that would require counter-gradient fluxes to obtain higher mixing ratios in the upper than in the lower troposphere^15^. Transport of Asian tracers to North America was faster in the upper troposphere and Asian tracers of 4–6 days in age were found at an altitude of 10 km in North America; in the case of tracer aged 8–10 days the maximum signal had descended to an altitude of 6–8 km^15^. This is consistent with previous findings that most of the tracer was transported at higher altitudes and later trapped in the quasi-permanent subtropical anticyclone west of North America, which eventually subsides.

However, in the present study, the CMAQ-RAMS and HYSPLIT back trajectory model results provided a comprehensive spatiotemporal representation of the transportation of PM_2.5_ that strongly supports the conclusion that ground-level transport could a the major transport pathway for pollutants at the scale of several kilometers in distance.

The findings of this study show that cold surges have the potential to transport air pollutants up to a distance of 2000 km or more in one or two days. This is the first study that explored long transport of PM_2.5_ between Beijing, Shanghai, and Xiamen. The maximum wind speed for cold surges was 25 m/s in Beijing, 20 m/s in Shanghai, and 22 m/s in Xiamen. The distance between Beijing and Xiamen is about 2000 km, and during the second cold surge, the wind speed in all the three cities reached 22 m/s. Compared to Shanghai and Xiamen, the maximum wind speeds of inland cities Taiyuan, Zhengzhou, and Hefei, were relatively lower but still exceeded 10 m/s during the two cold surges. Therefore, haze could travel with cold surges from Beijing to Xiamen within two days. A reliable assessment of PM_2.5_ concentrations is difficult due to various non-point pollution sources, which influence the physical, chemical and biological formation of PM_2.5_, and the processes involved in transport, deposition, suspension, resuspension, dispersion, dilution and secondary aerosol formation progresses. Given that the residence time of PM_2.5_ in the atmosphere is 3–5 days^42^, with wind speeds over 20 m/s, PM_2.5_ particles can be transported 2000 km from Beijing by cold surges to Xiamen within two days.

Air quality in Xiamen could be significantly influenced by meteorological conditions that facilitate the transport of air pollutants from northern China. In this study, we found that air pollutants from Beijing could be transported to Xiamen by strong cold surges within one or two days. While wind speed was found be a major contributor to air pollution during the winter in Xiamen, further research is required to establish the relationship between wind speed and PM_2.5_ transport in other geographical regions.

## Materials and Methods

### Air quality monitoring network in China

The third version of the “the national ambient air quality standards” (NAAQS) (GB3095-2012), implemented by the Ministry of Environmental Protection of China (MEP) in 2012, included PM_2.5_ for the first time. The Chinese government has started to construct a national air quality monitoring network following the implementation of NAAQS. As of 2015, the network consisted of 1665 stations in 366 cities. The real-time mass concentrations of PM_2.5_ and PM_10_ are measured using the oscillating microbalance method and/or the β absorption method. Instrumental operation, maintenance, data assurance and quality control were conducted based on the most recent revisions of China’s Environmental Protection Standards. Real-time hourly concentrations of PM_2.5_ and other major air pollutants (i.e. SO_2_, NO_2_, PM_2.5_ PM_10_, CO, and O_3_), were continuously recorded by the MEP in China and are publicly accessible from the MEP data center (http://datacenter.mep.gov.cn/).

Beijing (116.23°E, 40.30°N), Shanghai (121.51°E, 31.27°N), and Xiamen (118.17°E, 24.82°N) (Fig. 1) were selected as representative cities of north, central, and south China to investigate the impact of meteorological conditions, mainly wind field, on air quality. These cities were selected for this study based on their GDP and population size, which are economic proxies for PM_2.5_. Beijing is the political center of China with GDP 2133 billion RMB and population of 13.3 million in 2014, Shanghai is the economic megacity in central China with GDP 2356 billion RMB and population of 14.4 million in 2014, and Xiamen is a coastal city with high air quality with GDP 327 billion RMB and population of 4.0 million in 2014.

### Meteorological data

The 6-hourly wind data at ground level during 1–10 February 2015 was obtained from the European Centre for Medium-Range Weather Forecasts Re-Analysis Interim (ERA-Interim) on a 0.75° × 0.75° grid^43^ (http://apps.ecmwf.int/datasets/data/interim-full-daily/levtype=pl/). Meteorological data such as temperature, wind direction, and wind speed for Beijing, Shanghai, and Xiamen were downloaded from National Oceanic and Atmospheric Administration (NOAA) (https://www.climate.gov/maps-data).

### HYSPLIT back trajectory Model

In order to trace source and transport paths of air parcels arriving in Xiamen, the National Oceanic and Atmospheric Administration (NOAA) Hybrid Single Particle Lagrangian Integrated Trajectory (HYSPLIT) model was used to compute the 72-hour backward trajectory ending at 10 m height in Xiamen for the study interval. The HYSPLIT model is a complete system for computing simple air parcel trajectories, as well as complex transport, dispersion, chemical transformation, and deposition simulations, and is available on the NOAA Air Resources Laboratory website (http://www.arl.noaa.gov/ready.html). Reanalyzed archived data from the NCEP/NCAR was utilized in this study. The meteorological data used to initialize HYSPLIT was obtained from the NCEP Global Data Assimilation System (GDAS) with one-degree resolution. All model runs include available observations were produced within six-hourly analyses and short-range forecasts. Vertical velocities obtained from the continuity equation used in the GDAS model surfaces were vertically interpolated to the sigma coordinate system in the trajectory model.

### The RAMS-CMAQ model

The air quality modeling system, regional atmospheric modeling system, and community multi-scale air quality model (RAMS-CMAQ) were used to reproduce the effects of cold air surges on the concentration of PM_2.5_ in Beijing, Shanghai, and Xiamen^10,13,22,44^. CMAQ is an Eulerian-type model that concurrently simulates all atmospheric and land processes that affect the transport, transformation and deposition of air pollutants and their precursors, on both regional and urban scales.

RAMS provided the three-dimensional meteorological fields required for CMAQ. In this study, RAMS was implemented in a four-dimensional data assimilation mode using analysis nudging with re-initialization set to a time interval of four days, leaving the first 24 hours as the initialization period. The three-dimensional meteorological fields for RAMS were obtained from the National Centers for Environmental Prediction (NCEP) final analyses datasets (6-h intervals, 1° × 1° resolution). The study domain for CMAQ was 6654 × 5440 km^2^ on a rotated polar stereographic map projection centered at (35.0°N, 116.0°E), with a grid resolution of 64 × 64 km^2^. This region exhibited major variations in topography and land use, and was comprised of industrial areas, urban centers, and rural agricultural regions. RAMS and CMAQ have the same model height. For RAMS, there were 25 vertical layers in the σ_z_-coordinate system, unequally spaced from the ground to ~23 km, approximately nine layers of which were concentrated in the lowest 2 km of the atmosphere to resolve the planetary boundary layer. CMAQ used 15 vertical levels with the lowest seven layers being the same as those in RAMS. The data of 50 m extracted from the model was compared to the monitoring data.

### Graphic software

Figure 1 is plotted using MATLAB R2013a (http://www.mathworks.com/). Figures 2, 3 and 5 were generated by the NCAR Command Language (Version 6.4.0, [Software]. (2017). Boulder, Colorado: UCAR/NCAR/CISL/TDD. http://dx.doi.org/10.5065/D6WD3XH5). Figure 4 was plotted using HYSPLIT Trajectory Model provided by Air Resources Laboratory of NOAA^27,28^.

### Data and materials availability

All data needed to evaluate the conclusions in the paper are present in the paper and/or the Supplementary Materials.

## Electronic supplementary material

Supplemental Figures
