# Supplementary material for: Large-scale transport of PM2.5 in the lower troposphere during winter cold surges in China
Source: Sci Rep. 2017 Oct 16;7:13238. doi: 10.1038/s41598-017-13217-2 (PMC5643490; doi:10.1038/s41598-017-13217-2)

Supplementary information

Large-scale transport of $PM_{2.5}$ in the lower troposphere during winter cold surges in China

Jianjun Wang,[1] Meigen Zhang,[2] Xiaolin Bai,[3] Hongjian Tan,[1] Sabrina Li,[4] Jiping Liu,[5] Rui Zhang,[3] Mark A. Wolters,[6] Xiuyuan Qin,[1] Miming Zhang,[1] Hongmei Lin,[1] Yuenan Li,[6] Jonathan Li,[4] Liqi Chen[1]

1Key Laboratory of Global Change and Marine-Atmospheric Chemistry, Third Institute of Oceanography, State Oceanic Administration, Xiamen, China

2 State Key Laboratory of Atmospheric Boundary Layer Physics and Atmospheric Chemistry, Institute of Atmospheric Physics, Chinese Academy of Sciences, Beijing, China

3 State Key Laboratory of Marine Environmental Science, and College of Ocean & Earth Sciences, Xiamen University, Xiamen, China

4 Department of Geography and Environmental Management, University of Waterloo, Canada

5 Department of Atmospheric and Environmental Sciences, University at Albany, State University of New York, Albany, USA

6 Shanghai Center for Mathematical Sciences, Fudan University, Shanghai, China

Correspondence and requests for materials should be addressed to L.C. (email: chenliqi@tio.org.cn) or J.L. (email: junli@uwaterloo.ca)

Contents: The legend of the supplemental dataset and 3 figures

The legend of the supplemental dataset:

The animation of $PM_{2.5}$ and wind field during 1-10 February, 2015. The animation is made using MATLAB R2013a (http://www.mathworks.com/)

Figure R1 Back trajectories for Xiamen during 19:00 on 5th-23:00 on 6th and 09:00 on 8th -20:00 on 9th February, 2015. Figures are plotted using HYSPLIT Trajectory Model provided by Air Resources Laboratory of NOAA[29, 30].

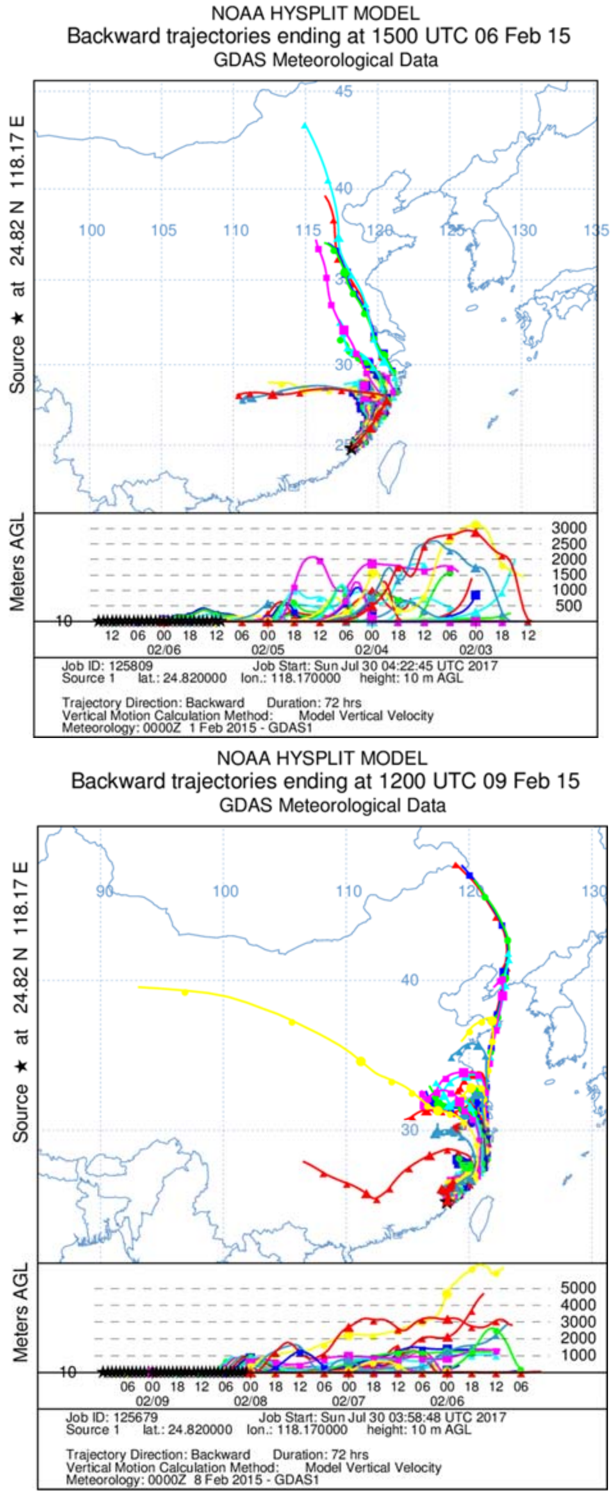

Figure S2 Temporal and spatial distribution of the wind field and concentrations of PM2.5 at 2122m during 1-10 February, 2015. The figures was generated by the NCAR Command Language (Version 6.4.0, [Software]. (2017). Boulder, Colorado: UCAR/NCAR/CISL/TDD. http://dx.doi.org/10.5065/D6WD3XH5).

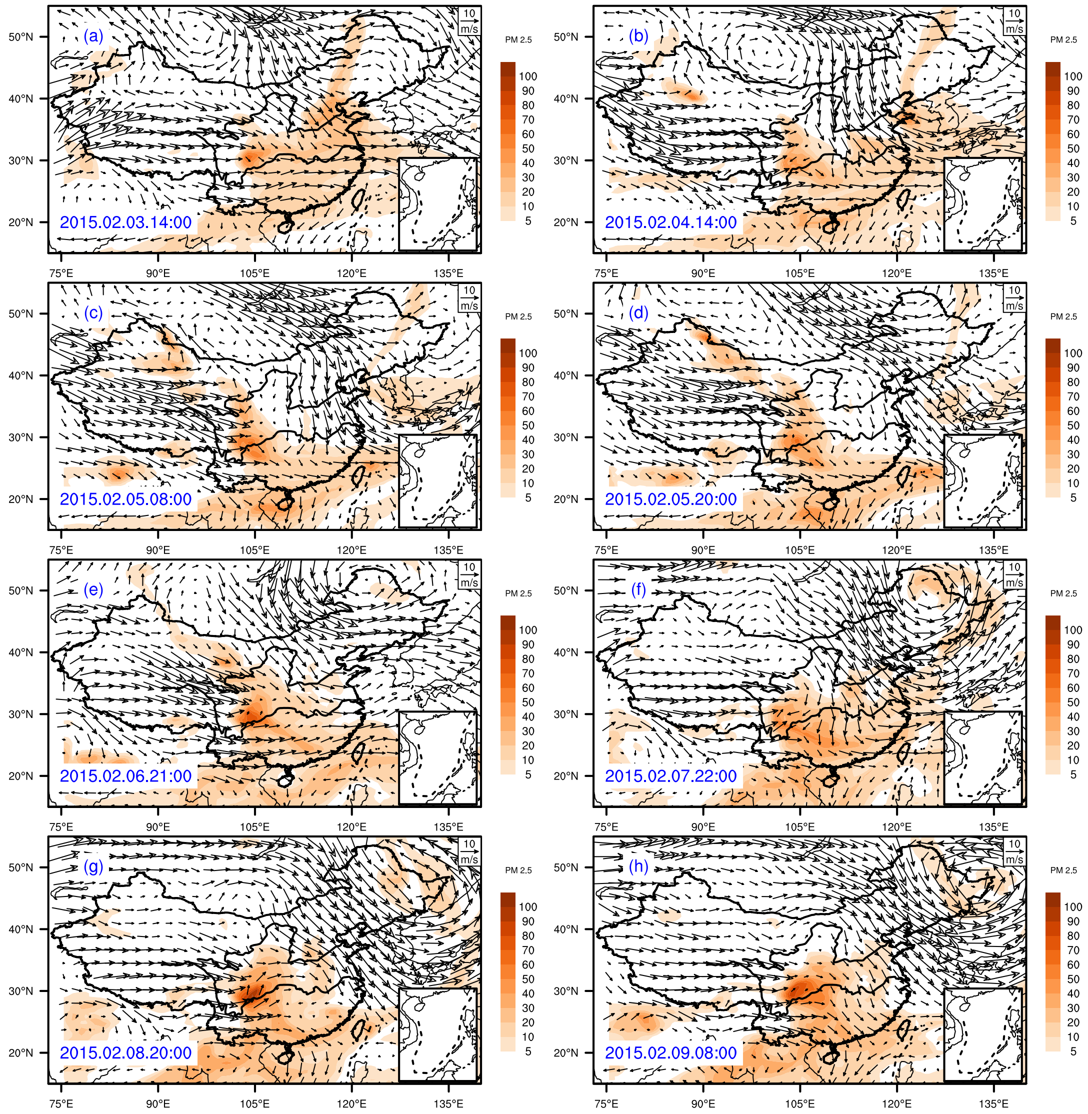

Figure S3 Temporal and spatial distribution of temperature at ground level during 1-10 February, 2015. The figures was generated by the NCAR Command Language (Version 6.4.0, [Software]. (2017). Boulder, Colorado: UCAR/NCAR/CISL/TDD. http://dx.doi.org/10.5065/D6WD3XH5).

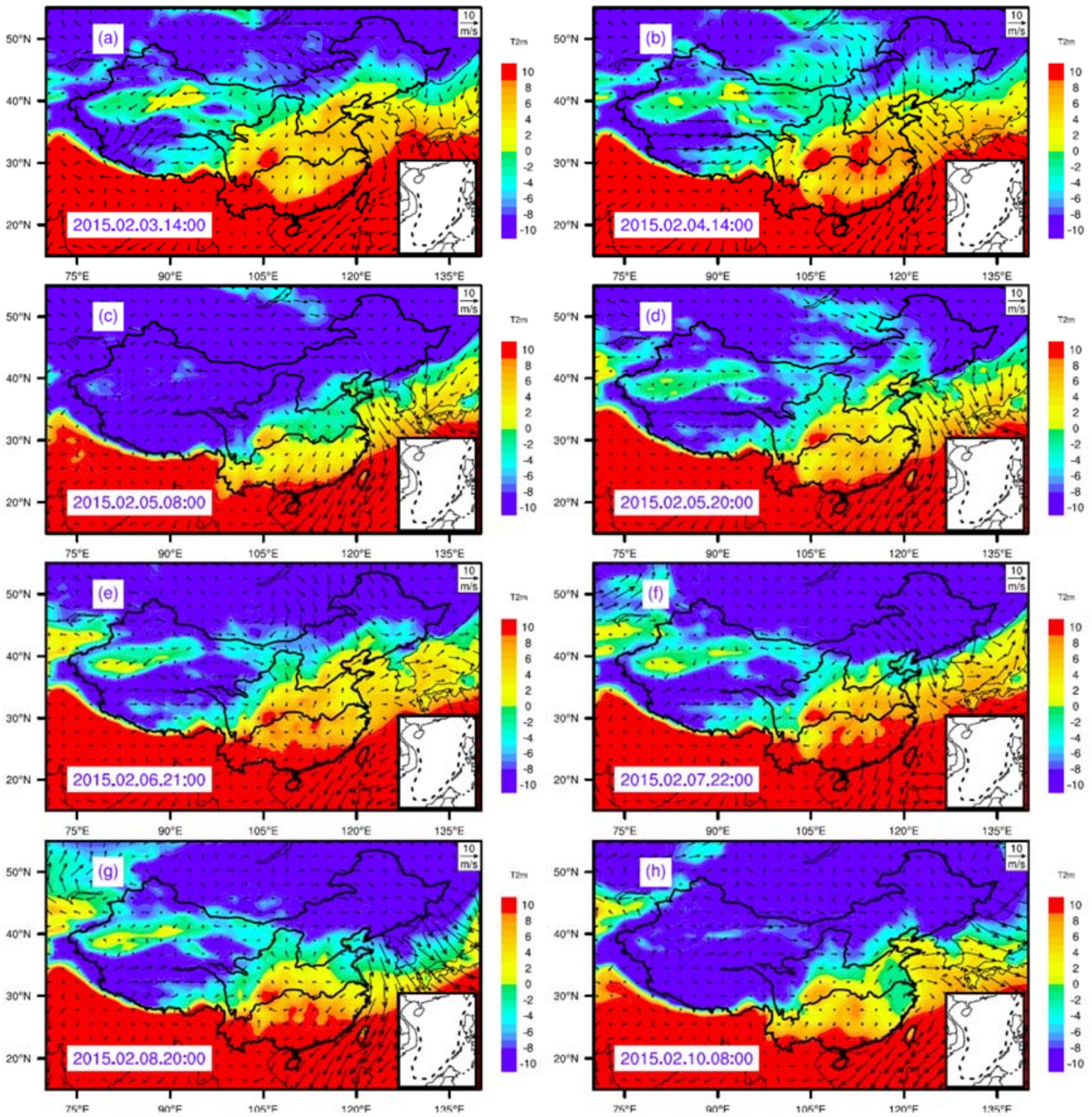

Supplement: Supplementary file 1 — Supplemental Figures [file 41598_2017_13217_MOESM1_ESM.pdf]
